# Tracheophyte of Xiao Hinggan Ling in China: an updated checklist

**DOI:** 10.3897/BDJ.7.e32306

**Published:** 2019-03-27

**Authors:** Hongfeng Wang, Xueyun Dong, Yi Liu, Keping Ma

**Affiliations:** 1 School of Forestry, Northeast Forestry University, Harbin, China School of Forestry, Northeast Forestry University Harbin China; 2 School of Food Engineering Harbin University, Harbin, China School of Food Engineering Harbin University Harbin China; 3 Environment Change Institute of Botany Chinese Academy of Sciences Nanxincun 20, Xiangshan, Beijing, China Environment Change Institute of Botany Chinese Academy of Sciences Nanxincun 20, Xiangshan Beijing China; 4 State Key Laboratory of Vegetation and Environmental Change, Institute of Botany, Chinese Academy of Sciences, Beijing, China State Key Laboratory of Vegetation and Environmental Change, Institute of Botany, Chinese Academy of Sciences Beijing China

**Keywords:** Xiao Hinggan Ling, tracheophyte, flora

## Abstract

**Background:**

This paper presents an updated list of tracheophytes of Xiao Hinggan Ling. The list includes 124 families, 503 genera and 1640 species (Containing subspecific units), of which 569 species (Containing subspecific units), 56 genera and 6 families represent first published records for Xiao Hinggan Ling. The aim of the present study is to document an updated checklist by reviewing the existing literature, browsing the website of National Specimen Information Infrastructure and additional data obtained in our research over the past ten years.

This paper presents an updated list of tracheophytes of Xiao Hinggan Ling. The list includes 124 families, 503 genera and 1640 species (Containing subspecific units), of which 569 species (Containing subspecific units), 56 genera and 6 families represent first published records for Xiao Hinggan Ling. The aim of the present study is to document an updated checklist by reviewing the existing literature, browsing the website of National Specimen Information Infrastructure and additional data obtained in our research over the past ten years.

**New information:**

This is the first complete checklist of tracheophytes of Xiao Hinggan Ling. A total of 1640 species and subspecies are listed, of which 569 species (ontaining subspecific units) represent first published records.

## Introduction

Xiao Hinggan Ling is located in the north of Heilongjiang Province, China. It is a part of the low mountains and lava platforms in northeast China. It is an important part of the Hinggan Ling in northeast Asia. This area intersects with the remnants of the Da Hinggan Ling, the Northeast Plain and the Changbaishan Mountains. The floristic composition of Xiao Hinggan Ling is complex, and the plant species are diverse in Xiao Hinggan Ling. Typical zonal vegetation types in the region are broad-leaved Korean pine mixed forest and Korean pine forest. The broad-leaved coniferous forest in the Xiao Hinggan Ling region of northeast in China is a very important timber resource (Cao and Li 2007). Xiao Hinggan Ling areas have the world's largest virgin forest of *Pinus koraiensis*, known as "The Home Of Korean Pine" (Wu et al. 2013).

The catalogue of tracheophyte published in 2007 (in “Floristics and Distribution of Plants in Xiao Hinggan Ling, China”) includes 1161 species belonging to 439 genera and 114 families (Cao and Li 2007). Among them, there are 59 species belonging to 30 genera and 14 families of clubmosses and ferns, 12 species belonging to 6 genera and 3 families of gymnosperms, and 1000 species belonging to 411 genera and 101 families of angiosperms. This old Checklist is mainly based on the specimen information and plant investigation and collection records of The Herbarium of Institute of Applied Ecology, Chinese Academy of Sciences (IFP). In recent years, China has built a unified platform, the National Specimen Resource Platform of China (NSII) (NSII 2016) integrating digital specimen information in the whole country. The number of plant specimens in NSII was about 24 times that of IFP, reaching 10.42 million. We collect all the tracheophyte specimen information from Xiao Hinggan Ling on NSII and other materials and surveyed the region for more than 10 years. Now we present a new checklist of tracheophyte in Xiao Hinggan Ling.

## Materials and methods

The data presented in the checklist are based primarily on the results of field survey for more than 10 years and published information concerning NSII, field plant survey (a list of records and plant photos), “Floristics and distribution of plants in Xiao Hinggan Ling, China” (Cao and Li 2007), The International Plant Names Index (IPNI) (http://www.ipni.org/index.html) and Global Biodiversity Information Facility (GBIF) (GBIF 2014), the newl recorded and new species are obtained from the published taxonomic articles in recent ten years (e.g., Zheng and Cao 2013, Zheng and Pan 2012, Ma 2013, Ma 2014, Wang et al. 2013), and Flora of China (FOC 2013), Species Catalogue of China (Zhu et al. 2015). Scientific name in the original data were transformed into the accepted name in the The Plant List (TPL database) (http://www.theplantlist.org/1.1/about) by Taxonomic Name Resolution Service (TNRS) (http://tnrs.iplantcollaborative.org). Finally, we unified the classification system. PPGI system was used for Pteridophyte (PPG I 2016), Christenhusz system was used for gymnosperms (Christenhusz et al. 2010, Zhang 2017), and APGⅥ system was used for angiosperms (APG IV 2016) (Suppl. material 1).

## Checklists

### Newly recorded tracheophytes in Xiao Hinggan Ling

#### Pteridophyta

#### Polypodiales

#### Athyriaceae

#### Athyrium fallaciosum

Milde

#### Onocleaceae

#### Onoclea sensibilis

L.

#### Dennstaedtiaceae

#### Pteridium aquilinum

(L.) Kuhn

#### Equisetales

#### Equisetales

#### Equisetaceae

#### Equisetum ramosissimum

Desf.

#### Ophioglossales

#### Ophioglossaceae

#### Ophioglossum vulgatum

L.

#### Ophioglossum thermale

Kom.

#### Polypodiales

#### Dryopteridaceae

#### Dryopteris austriaca

(Jacq.) Woyn. ex Schinz & Thell.

#### Salviniales

#### Salviniaceae

#### Salvinia natans

(L.) All.

#### Gymnospermae

#### Pinales

#### Pinaceae

#### Abies holophylla

Maxim.

#### Larix gmeliniivar.principis-rupprechtii

(Mayr) Pilg.

#### Picea koyamae

Shiras.

#### Picea obovata

Ledeb.

#### Pinus armandii

Franch.

#### Pinus densiflora

Siebold & Zucc.

#### Angiospermae

#### Acorales

#### Acoraceae

#### Acorus calamus

L.

#### Alismatales

#### Alismataceae

#### Alisma gramineum

Lej.

#### Sagittaria trifolia

L.

#### Butomaceae

#### Butomus umbellatus

L.

#### Hydrocharitaceae

#### Najas minor

All.

#### Potamogetonaceae

#### Potamogeton octandrusvar.mizuhikimo

(Makino) H.Hara

#### Potamogeton oxyphyllus

Miq.

#### Potamogeton perfoliatus

L.

#### Potamogeton polygonifolius

Pourr.

#### Potamogeton pusillus

L.

#### Apiales

#### Apiaceae

#### Anethum graveolens

L.

#### Cnidium dauricum

(Jacq.) Fisch. & C.A.Mey.

#### Eriocycla albescensvar.latifolia

Shan et Yuan

#### Osmorhiza aristata

Makino & Jabe

#### Ostericum sieboldiivar.praeteritum

(Kitag.) Y. Huei Huang

#### Pleurospermum uralense

Hoffm.

#### Angelica amurensis

Schischk.

#### Angelica cartilaginomarginata

(Makino ex Y.Yabe) Nakai

#### Angelica decursiva

(Miq.) Franch. & Sav.

#### Angelica gigas

Nakai

#### Angelica nakaiana

(Kitag.) Pimenov

#### Angelica polymorpha

Maxim.

#### Anthriscus sylvestris

(L.) Hoffm.

#### Bupleurum angustissimum

(Franch.) Kitag.

#### Bupleurum bicaule

Helm

#### Carlesia sinensis

Dunn

#### Heracleum hemsleyanum

Diels

#### Heracleum lanatum

Michx.

#### Oenanthe javanica rosthornii

(Diels) F.T. Pu

#### Peucedanum terebinthaceumvar.deltoideum

(Makino ex K. Yabe) Makino

#### Sanicula tuberculata

Maxim.

#### Araliaceae

#### Aralia continentalis

Kitag.

#### Panax pseudoginseng

Wall.

#### Panax quinquefolius

L.

#### Asparagales

#### Amaryllidaceae

#### Allium anisopodium

Ledeb.

#### Allium leucocephalum

Turcz. ex Ledeb.

#### Allium przewalskianum

Regel

#### Allium sacculiferum

Maxim.

#### Allium schoenoprasum

L.

#### Allium thunbergii

G.Don

#### Asparagaceae

#### Asparagus dauricus

Fisch. ex Link

#### Asparagus trichophyllus

Bunge

#### Convallaria majalis

L.

#### Polygonatum odoratumvar.pluriflorum

(Miq.) Ohwi

#### Polygonatum odoratumvar.thunbergii

(C.Morren & Decne.) H.Hara

#### Polygonatum stenophyllum

Maxim.

#### Hemerocallis citrina

Baroni

#### Hemerocallis fulva

(L.) L.

#### Iridaceae

#### Iris setosa

Pall. ex Link

#### Iris ventricosa

Pall.

#### Orchidaceae

#### Epipactis xanthophaea

Schltr.

#### Epipactis helleborine

(L.) Crantz

#### Cypripedium shanxiense

S. C. Chen

#### Habenaria linearifolia

Maxim.

#### Asterales

#### Asteraceae

#### Nabalus tatarinowii

(Maxim.) Nakai

#### Ligularia sachalinensis

Nakai

#### Parasenecio firmus

(Kom.) Y.L.Chen

#### Parasenecio hastatusvar.glaber

(Ledeb.) Y.L.Chen

#### Picris hieracioides japonica

(Thunb.) Krylov

#### Saussurea chinnampoensis

H.Lév. & Vaniot

#### Saussurea elongata

DC.

#### Saussurea maximowiczii

Herder

#### Saussurea sclerolepis

Nakai & Kitag.

#### Saussurea stoliczkae

C.B.Clarke

#### Saussurea tenerifolia

Kitag.

#### Saussurea tomentosa

Kom.

#### Saussurea ussuriensis

Maxim.

#### Scorzonera austriaca

Willd.

#### Senecio ambraceus

Turcz. ex DC.

#### Senecio cannabifoliusvar.integrifolius

(Koidz.) Kitam.

#### Serratula cupuliformis

Nakai & Kitag.

#### Serratula marginata

Tausch

#### Serratula polycephala

Iljin

#### Solidago decurrens

Lour.

#### Sonchus transcaspicus

Nevski

#### Taraxacum asiaticum

Dahlst.

#### Taraxacum mongolicum

Hand.-Mazz.

#### Taraxacum platypecidum

Diels

#### Taraxacum sinicum

Kitag.

#### Tephroseris kirilowii

(Turcz. ex DC.) Holub

#### Tephroseris palustris

(L.) Rchb.

#### Tephroseris phaeantha

(Nakai) C.Jeffrey & Y.L.Chen

#### Tripleurospermum limosum

(Maxim.) Pobed.

#### Asterales

#### Campanulaceae

#### Adenophora capillaris paniculata

(Nannf.) D.Y.Hong & S.Ge

#### Adenophora grandiflora

Nakai

#### Adenophora palustris

Kom.

#### Adenophora polyantha

Nakai

#### Adenophora stenanthina

(Ledeb.) Kitag.

#### Adenophora stenophylla

Hemsl.

#### Campanula glomerata speciosa

(Hornem. ex Spreng.) Domin

#### Boraginales

#### Boraginaceae

#### Amblynotus rupestris

(Pall.) Popov

#### Eritrichium borealisinense

Kitag.

#### Myosotis laxa caespitosa

(Schultz) Hyl. ex Nordh.

#### Trigonotis peduncularisvar.amblyosepala

(Nakai & Kitag.) W.T. Wang

#### Arabidopsis lyrata kamchatica

(Fisch. ex DC.) O'Kane & Al-Shehbaz

#### Arabidopsis halleri

(L.) O'Kane & Al-Shehbaz

#### Stevenia cheiranthoides

DC.

#### Cardamine flexuosa

With.

#### Cardamine komarovii

Nakai

#### Cardamine parviflora

L.

#### Cardamine pratensis

L.

#### Dontostemon hispidus

Maxim.

#### Dontostemon micranthus

C.A.Mey.

#### Erysimum perofskianum

Fisch. & C.A.Mey.

#### Erysimum flavum

(Georgi) Bobrov

#### Lepidium apetalum

Willd.

#### Lepidium densiflorum

Schrad.

#### Caryophyllales

#### Amaranthaceae

#### Corispermum macrocarpum

Bunge ex Maxim.

#### Corispermum elongatum

Bunge ex Maxim.

#### Amaranthus albus

L.

#### Amaranthus retroflexus

L.

#### Dysphania botrys

(L.) Mosyakin & Clemants

#### Caryophyllaceae

#### Stellaria cherleriae

(Fisch. ex Ser.) F.N. Williams

#### Stellaria bungeana

Fenzl

#### Cerastium arvense

L.

#### Dianthus repens

Willd.

#### Dianthus superbus

L.

#### Eremogone capillaris

(Poir.) Fenzl

#### Gypsophila oldhamiana

Miq.

#### Lychnis wilfordii

(Regel) Maxim.

#### Minuartia macrocarpavar.koreana

(Nakai) Hara

#### Pseudostellaria rupestris

(Turcz.) Pax

#### Silene baccifera

(L.) Roth

#### Silene foliosa

Maxim.

#### Silene jeniseensis

Willd.

#### Silene nocturnavar.brachypetala

(Robill. & Castagne ex DC.) Benth.

#### Stellaria graminea

L.

#### Plumbaginaceae

#### Limonium bicolor

(Bunge) Kuntze

#### Polygonaceae

#### Atraphaxis manshurica

Kitag.

#### Atraphaxis frutescens

(L.) K.Koch

#### Fallopia dentatoalata

(F.Schmidt) Holub

#### Persicaria maculosa

Gray

#### Persicaria vivipara

(L.) Ronse Decr.

#### Polygonum avicularevar.fusco-ochreatum

(Kom.) A.J.Li

#### Polygonum boreale

(Lange) Small

#### Polygonum foliosumvar.paludicola

(Makino) Kitam.

#### Polygonum kawagoeanum

Makino

#### Polygonum lapathifoliumvar.lanatum

(Roxb.) Steward

#### Polygonum maackianum

Regel

#### Polygonum muricatum

Meisn.

#### Polygonum ochreatum

L.

#### Polygonum patulum

M.Bieb.

#### Polygonum posumbu

Buch.-Ham. ex D. Don

#### Polygonum rigidum

Skvortsov

#### Polygonum sibiricum

Laxm.

#### Polygonum trigonocarpum

(Makino) Kudô & Masam.

#### Rumex aquaticus

L.

#### Rumex crispus

L.

#### Rumex marschallianus

Rchb.

#### Rumex patientia

L.

#### Rumex stenophyllus

Ledeb.

#### Celastrales

#### Celastraceae

#### Celastrus orbiculatus

Thunb.

#### Euonymus hamiltonianus

Wall.

#### Euonymus oxyphyllus

Miq.

#### Euonymus rehderianus

Loes.

#### Parnassia palustrisvar.multiseta

Ledeb.

#### Ceratophyllales

#### Ceratophyllaceae

#### Ceratophyllum demersum

L.

#### Commelinales

#### Linaceae

#### Linum baicalense

Juz.

#### Linum perenne

L.

#### Linum usitatissimum

L.

#### Cornales

#### Cornaceae

#### Alangium platanifolium

(Siebold & Zucc.) Harms

#### Hydrangeaceae

#### Deutzia baroniana

Diels

#### Deutzia parviflora

Bunge

#### Dioscoreales

#### Dioscoreaceae

#### Dioscorea oppositifolia

L.

#### Dipsacales

#### Adoxaceae

#### Sambucus latipinna

Nakai

#### Sambucus sibirica

Nakai

#### Viburnum mongolicum

(Pall.) Rehder

#### Caprifoliaceae

#### Lonicera caerulea

L.

#### Lonicera ferdinandii

Franch.

#### Lonicera japonica

Thunb.

#### Lonicera nigra

L.

#### Lonicera subhispida

Nakai

#### Patrinia scabra

Bunge

#### Patrinia scabiosifolia

Link

#### Ericales

#### Balsaminaceae

#### Impatiens furcillata

Hemsl.

#### Polemonium caeruleum

L.

#### Primulaceae

#### Androsace septentrionalis

L.

#### Lysimachia clethroides

Duby

#### Primula farinosa

L.

#### Primula matthioli

(L.) K.Richt.

#### Primula patens

E.A. Busch

#### Primula pumilio

Maxim.

#### Primula saxatilis

Kom.

#### Fagales

#### Betulaceae

#### Carpinus cordata

Blume

#### Ostryopsis davidiana

Decne.

#### Betula chinensis

Maxim.

#### Betula ovalifolia

Rupr.

#### Betula schmidtii

Regel

#### Fabaceae

#### Melilotus dentatus

(Waldst. & Kit.) Pers.

#### Glycyrrhiza pallidiflora

Maxim.

#### Hedysarum vicioides

Turcz.

#### Hylodesmum oxyphyllum

(DC.) X.F. Gao

#### Melilotus albus

Medik.

#### Oxytropis hailarensis

Kitag.

#### Astragalus chinensis

L.f.

#### Astragalus complanatus

Bunge

#### Astragalus filiformis

(DC.) Poir.

#### Astragalus galactites

Pall.

#### Astragalus monbeigii

G.Simpson

#### Astragalus penduliflorus

Lam.

#### Desmodium oldhamii

Oliv.

#### Glycine max

(L.) Merr.

#### Lathyrus quinquenervius

(Miq.) Litv.

#### Lathyrus vaniotii

H.Lev.

#### Lespedeza cyrtobotrya

Miq.

#### Lespedeza floribunda

Bunge

#### Trifolium hybridum

L.

#### Trifolium pratense

L.

#### Trifolium repens

#### Vicia cracca

L.

#### Vicia multicaulis

Ledeb.

#### Vicia ohwiana

Hosok.

#### Vicia venosa

(Link) Maxim.

#### Vicia tenuifolia

Roth

#### Quercus dentata

Thunb.

#### Quercus wutaishanica

Mayr

#### Gentianales

#### Apocynaceae

#### Periploca sepium

Bunge

#### Cynanchum acutum sibiricum

(Willd.) Rech.f.

#### Gentianaceae

#### Gentiana caeruleogrisea

T.N.Ho

#### Gentiana scabravar.buergeri

(Miq.) Maxim. ex Franch. & Sav.

#### Gentiana zollingeri

Fawc.

#### Swertia perennis

L.

#### Rubiaceae

#### Galium platygalium

(Maxim.) Pobed.

#### Galium rubioides

L.

#### Galium spurium

L.

#### Galium trifloriforme

Kom.

#### Geraniales

#### Geraniaceae

#### Geranium dahuricumvar.paishanense

(Y.L.Chang) C.C.Huang & L.R.Xu

#### Geranium erianthum

DC.

#### Geranium koreanum

Kom.

#### Geranium krameri

Franch. & Sav.

#### Geranium transbaicalicum

Serg.

#### Geranium wilfordii

Maxim.

#### Lamiales

#### Lamiaceae

#### Phlomoides umbrosa

(Turcz.) Kamelin & Makhm.

#### Phlomoides maximowiczii

(Regel) Kamelin & Makhm.

#### Clinopodium chinense

(Benth.) Kuntze

#### Dracocephalum moldavica

L.

#### Dracocephalum ruyschiana

L.

#### Elsholtzia densa

Benth.

#### Elsholtzia splendens

Nakai ex F.Maek.

#### Isodon inflexus

(Thunb.) Kudô

#### Lamium album barbatum

(Siebold & Zucc.) Mennema

#### Leonurus macranthus

Maxim.

#### Leonurus sibiricus

L.

#### Leonurus sibiricus

L.

#### Lycopus lucidusvar.hirtus

Regel

#### Scutellaria moniliorhiza

Kom.

#### Scutellaria pekinensis

Maxim.

#### Scutellaria regelianavar.ikonnikovii

(Juz.) C.Y.Wu & H.W.Li

#### Scutellaria strigillosa

Hemsl.

#### Stachys riederivar.hispidula

(Regel) H.Hara

#### Stachys riederivar.japonica

(Miq.) H.Hara

#### Linderniaceae

#### Lindernia procumbens

(Krock.) Philcox

#### Oleaceae

#### Syringa pubescens patula

(Palib.) M.C.Chang & X.L.Chen

#### Orobanchaceae

#### Euphrasia amurensis

Freyn

#### Euphrasia pectinata simplex

(Freyn) Hong

#### Pedicularis sceptrum-carolinum pubescens

(Bunge) Tsoong

#### Pedicularis verticillata

L.

#### Phrymaceae

#### Phryma leptostachyavar.oblongifolia

(Koidz.) Honda

#### Plantaginaceae

#### Trapella sinensis

Oliv.

#### Callitriche hermaphroditica

L.

#### Linaria vulgaris

Mill.

#### Plantago depressa turczaninowii

(Ganesch.) Tzvelev

#### Pseudolysimachion linariifolium dilatatum

(Nakai & Kitag.) D.Y. Hong

#### Veronica daurica

Steven

#### Veronica rotunda

Nakai

#### Veronica spicata incana

(L.) Walters

#### Veronica stelleri

Pall. ex Link

#### Liliales

#### Liliaceae

#### Gagea pauciflora

(Turcz. ex Trautv.) Ledeb.

#### Gagea triflora

(Ledeb.) Schult. & Schult.f.

#### Lilium browniivar.viridulum

Baker

#### Lilium cernuum

Kom.

#### Lilium concolor

Salisb.

#### Smilacaceae

#### Smilax nipponica

Miq.

#### Smilax riparia

A.DC.

#### Malpighiales

#### Ericaceae

#### Arctous rubra

(Rehder & E.H.Wilson) Nakai

#### Arctous alpina

(L.) Nied.

#### Monotropastrum humile

(D.Don) H.Hara

#### Pyrola japonica

Klenze ex Alef.

#### Pyrola renifolia

Maxim.

#### Pyrola rotundifolia

L.

#### Pyrola tschanbaischanica

Y.L. Chou & Y.L. Chang

#### Rhododendron micranthum

Turcz.

#### Rhododendron mucronulatum

Turcz.

#### Euphorbiaceae

#### Euphorbia esulavar.cyparissioides

Boiss.

#### Euphorbia fischeriana

Steud.

#### Euphorbia hirta

L.

#### Euphorbia maculata

L.

#### Euphorbia pekinensis

Rupr.

#### Euphorbia pilosa

L.

#### Euphorbia sieboldiana

C.Morren & Decne.

#### Euphorbia thymifolia

L.

#### Phyllanthaceae

#### Phyllanthus ussuriensis

Rupr. & Maxim.

#### Phyllanthus urinaria

L.

#### Salicaceae

#### Populus simonii

Carrière

#### Salix bebbiana

Sarg.

#### Salix caspica

Pall.

#### Salix forrestii

K.S.Hao

#### Salix microstachya

Turcz.

#### Salix miyabeana

Seemen

#### Salix rotundifolia

Trautv.

#### Salix starkeana

Willd.

#### Violaceae

#### Viola bangii

Rusby

#### Viola biflora

L.

#### Viola philippica

Cav.

#### Viola prionantha

Bunge

#### Viola rossii

Hemsl.

#### Myrtales

#### Lythraceae

#### Trapa natansvar.bispinosa

(Roxb.) Makino

#### Trapa natans

L.

#### Malvaceae

#### Grewia bilobavar.parviflora

(Bunge) Hand.-Mazz.

#### Tilia mongolica

Maxim.

#### Tilia platyphyllos

Scop.

#### Onagraceae

#### Oenothera villosa

Thunb.

#### Oenothera biennis

L.

#### Epilobium amurense

Hausskn.

#### Epilobium angustifolium circumvagum

Mosquin

#### Epilobium ciliatum

Raf.

#### Epilobium minutiflorum

Hausskn.

#### Nymphaeales

#### Nymphaeaceae

#### Euryale ferox

Salisb.

#### Nymphaea nouchali

Burm.f.

#### Oxalidales

#### Oxalidaceae

#### Oxalis griffithii

Edgew. & Hook. f.

#### Oxalis stricta

L.

#### Piperales

#### Araceae

#### Lemna japonica

Landolt

#### Lemna turionifera

Landolt

#### Aristolochiaceae

#### Aristolochia manshuriensis

Kom.

#### Poales

#### Cyperaceae

#### Trichophorum pumilum

(Vahl) Schinz & Thell.

#### Bolboschoenus planiculmis

(F.Schmidt) T.V.Egorova

#### Bolboschoenus yagara

(Ohwi) Y.C.Yang & M.Zhan

#### Carex breviculmis

R.Br.

#### Carex callitrichosvar.nana

(H.Lév. & Vaniot) S.Yun Liang, L.K.Dai & Y.C.Tang

#### Carex gotoi

Ohwi

#### Carex jaluensis

Kom.

#### Carex kirganica

Kom.

#### Carex kobomugi

Ohwi

#### Carex korshinskyi

Kom.

#### Carex lanceolatavar.laxa

Ohwi

#### Carex lanceolatavar.subpediformis

Kük.

#### Carex lithophila

Turcz.

#### Carex maackii

Maxim.

#### Carex mollicula

Boott

#### Carex raddei

Kük.

#### Carex sedakowii

C.A.Mey. ex Meinsh.

#### Carex siderostictavar.pilosa

H.Lév. ex T.Koyama

#### Carex xyphium

Kom.

#### Cyperus amuricus

Maxim.

#### Cyperus microiria

Steud.

#### Eleocharis acicularis

(L.) Roem. & Schult.

#### Eleocharis geniculata

(L.) Roem. & Schult.

#### Eleocharis maximowiczii

Zinserl.

#### Eleocharis retroflexa chaetaria

(Roem. & Schult.) T.Koyama

#### Fimbristylis squarrosa

Vahl

#### Fimbristylis squarrosavar.esquarrosa

Makino

#### Pycreus flavidusvar.nilagiricus

(Hochst. ex Steud.) Karthik.

#### Schoenoplectiella juncoides

(Roxb.) Lye

#### Scirpus lushanensis

Ohwi

#### Scirpus sylvaticus

L.

#### Eriocaulaceae

#### Eriocaulon atrum

Nakai

#### Eriocaulon decemflorum

Maxim.

#### Juncaceae

#### Juncus articulatus

L.

#### Juncus grisebachii

Buchenau

#### Oleaceae

#### Fraxinus bungeana

A.DC.

#### Fraxinus chinensis

Roxb.

#### Fraxinus chinensis rhynchophylla

(Hance) A.E.Murray

#### Poaceae

#### Avena sativa

L.

#### Cleistogenes polyphylla

Keng f.

#### Arthraxon hispidus

(Thunb.) Makino

#### Avena fatua

L.

#### Cleistogenes caespitosa

Keng

#### Lolium temulentum

L.

#### Agrostis divaricatissima

Mez

#### Agrostis macrathera

Phil.

#### Alopecurus longiaristatus

Maxim.

#### Alopecurus magellanicus

Lam.

#### Brachypodium sylvaticum

(Huds.) P.Beauv.

#### Bromus richardsonii

Link

#### Calamagrostis emodensis

Griseb.

#### Echinochloa oryzoides

(Ard.) Fritsch

#### Elymus gmeliniivar.macratherus

(Ohwi) S.L. Chen & G.H. Zhu

#### Elymus kamoji

(Ohwi) S. L. Chen

#### Eragrostis minor

Host

#### Festuca japonica

Makino

#### Festuca ovina

L.

#### Festuca parvigluma

Steud.

#### Glyceria lithuanica

(Gorski) Gorski

#### Hordeum vulgare

L.

#### Pennisetum flaccidum

Griseb.

#### Pennisetum glaucum

(L.) R.Br.

#### Poa acroleuca

Steud.

#### Puccinellia kobayashii

Ohwi

#### Typhaceae

#### Sparganium erectum

L.

#### Sparganium eurycarpum coreanum

(H.Lév.) C.D.K.Cook & M.S.Nicholls

#### Typha minima

Funck

#### Typha przewalskii

Skvortsov

#### Ranunculales

#### Asteraceae

#### Matricaria chamomilla

L.

#### Conyza japonica

(Thunb.) Less. ex Less.

#### Achillea millefolium

#### Ainsliaea acerifolia

Sch.Bip.

#### Artemisia aurata

Kom.

#### Artemisia brachyloba

Franch.

#### Artemisia brachyphylla

Kitam.

#### Artemisia kawakamii

Hayata

#### Artemisia laciniata

Willd.

#### Artemisia lancea

Vaniot

#### Artemisia oxycephala

Kitag.

#### Artemisia sylvatica

Maxim.

#### Artemisia vestita

Wall. ex Besser

#### Artemisia zhongdianensis

Y.R.Ling

#### Aster alpinus

L.

#### Aster hispidus

Thunb.

#### Atractylodes koreana

(Nakai) Kitam.

#### Atractylodes lancea

(Thunb.) DC.

#### Atractylodes ovata

(Thunb.) DC.

#### Bidens cernua

L.

#### Bidens parviflora

Willd.

#### Bidens tripartita

L.

#### Carpesium macrocephalum

Franch. & Sav.

#### Chrysanthemum indicum

L.

#### Chrysanthemum morifolium

Ramat.

#### Chrysanthemum zawadskii

Herbich

#### Cirsium shansiense

Petr.

#### Dendranthema lavandulifoliumvar.seticuspe

(Maxim.) C.Shih

#### Erigeron annuus

(L.) Pers.

#### Erigeron canadensis

L.

#### Erigeron thunbergii

A.Gray

#### Eupatorium lindleyanum

DC.

#### Kalimeris altaicusvar.millefolius

(Vant.) Wang

#### Hieracium hololeion

Maxim.

#### Inula britanicavar.sublanata

Komar.

#### Inula chinensis

(Kom.) Kom.

#### Inula salicina

L.

#### Jacobaea argunensis

(Turcz.) B.Nord.

#### Berberidaceae

#### Epimedium koreanum

Nakai

#### Papaveraceae

#### Corydalis buschii

Nakai

#### Corydalis lineariloba

Siebold & Zucc.

#### Corydalis repens

Mandl & Muhldorf

#### Corydalis speciosa

Maxim. ex Regel

#### Corydalis yanhusuo

(Y.H.Chou & Chun C.Hsu) W.T.Wang ex Z.Y.Su & C.Y.Wu

#### Hylomecon vernalis

Maxim.

#### Ranunculaceae

#### Batrachium kauffmanii

(Clerc) Krecz.

#### Leptopyrum fumarioides

(L.) Rchb.

#### Aconitum jaluense

Kom.

#### Aconitum kusnezoffiivar.gibbiferum

(Rchb.) Regel

#### Aconitum longecassidatum

Nakai

#### Aconitum monanthum

Nakai

#### Aconitum paniculigerum

Nakai

#### Aconitum septentrionale

Koelle

#### Aconitum tschangbaischanense

S.H.Li & Y.H.Huang

#### Adonis ramosa

Franch.

#### Anemone cathayensis

Kitag.

#### Anemone hepaticavar.japonica

(Nakai) Ohwi

#### Anemone raddeana

Regel

#### Anemone stolonifera

Maxim.

#### Anemone umbrosa

C.A.Mey.

#### Aquilegia flabellata

Siebold & Zucc.

#### Aquilegia viridifloravar.atropurpurea

(Willd.) Trevir.

#### Aquilegia yabeana

Kitag.

#### Caltha palustris

L.

#### Clematis aethusifolia

Turcz.

#### Clematis heracleifolia

DC.

#### Clematis intricata

Bunge

#### Clematis macropetala

Ledeb.

#### Clematis macropetalavar.albiflora

(Maxim. ex Kuntze) Hand.-Mazz.

#### Clematis nobilis

Nakai

#### Clematis pinnata

Maxim.

#### Clematis serratifolia

Rehder

#### Pulsatilla turczaninovii

Krylov & Serg.

#### Ranunculus cuneifolius

Maxim.

#### Ranunculus japonicusvar.hsinganensis

(Kitag.) W.T. Wang

#### Ranunculus monophyllus

Ovcz.

#### Ranunculus smirnovii

Ovcz.

#### Ranunculus trichophyllus eradicatus

(Laest.) C.D.K.Cook

#### Thalictrum filamentosum

Maxim.

#### Thalictrum foetidum

L.

#### Thalictrum minus

L.

#### Thalictrum petaloideum

L.

#### Thalictrum simplexvar.affine

(Ledeb.) Regel

#### Trollius asiaticus

L.

#### Trollius japonicus

Miq.

#### Trollius ledebourii

Rchb.

#### Rhamnaceae

#### Rhamnus arguta

Maxim.

#### Rhamnus diamantiaca

Nakai

#### Rhamnus parvifolia

Bunge

#### Rosales

#### Cannabaceae

#### Cannabis sativa

L.

#### Moraceae

#### Morus alba

L.

#### Rosaceae

#### Sibbaldia adpressa

Bunge

#### Sibbaldianthe bifurca

(L.) Kurtto & T.Erikss.

#### Filipendula glaberrima

Nakai

#### Geum macrophyllum

Willd.

#### Malus mandshurica

(Maxim.) Kom. ex Juz.

#### Potentilla acaulis

L.

#### Potentilla ancistrifoliavar.dickinsii

(Franch. & Sav.) Koidz.

#### Potentilla centigrana

Maxim.

#### Potentilla flagellaris

Willd. ex Schltdl.

#### Potentilla kleiniana

Wight & Arn.

#### Potentilla rosulifera

H.Lâ€šv.

#### Potentilla simulatrix

Th.Wolf

#### Potentilla supina

L.

#### Potentilla supinavar.ternata

Peterm.

#### Prunus armeniaca

L.

#### Prunus humilis

Bunge

#### Prunus padusvar.pubescens

Regel & Tiling

#### Prunus salicina

Lindl.

#### Prunus serrulata

Lindl.

#### Prunus wilsonii

(C.K.Schneid.) Koehne

#### Rubus chamaemorus

L.

#### Rubus crataegifolius

Bunge

#### Rubus humilifolius

C.A. Meyer

#### Rubus idaeus melanolasius

Dieck ex Focke

#### Rubus idaeus strigosus

(Michx.) Focke

#### Rubus parvifolius

L.

#### Sanguisorba canadensis

L.

#### Sanguisorba officinalisvar.longifila

(Kitag.) T.T.Yu & C.L.Li

#### Sanguisorba × tenuifoliavar.grandiflora

Maxim.

#### Sorbaria sorbifoliavar.stellipila

Maxim.

#### Sorbus alnifoliavar.lobulata

(Koidz.) Rehder

#### Spiraea dahurica

(Rupr.) Maxim.

#### Spiraea pubescens

Turcz.

#### Spiraea trichocarpa

Nakai

#### Ulmaceae

#### Ulmus davidiana

Planch.

#### Ulmus parvifolia

Jacq.

#### Ulmus pumila

L.

#### Urticaceae

#### Parietaria debilis

G.Forst.

#### Laportea cuspidata

(Wedd.) Friis

#### Urtica urens

L.

#### Santalales

#### Santalaceae

#### Thesium refractum

C.A.Mey.

#### Sapindaceae

#### Acer negundo

L.

#### Acer truncatum

Bunge

#### Saxifragales

#### Crassulaceae

#### Hylotelephium spectabile

(Boreau) H. Ohba

#### Phedimus hsinganicus

(Y.C. Chu ex S.H. Fu & Y. Hui Huang) H. Ohba, K.T. Fu & B.M. Barthol.

#### Sedum fedtschenckoi

Raym.-Hamet

#### Sedum kamtschaticum

Fisch.

#### Sedum pallescens

Freyn

#### Sedum roseum

(L.) Scop.

#### Sedum telephium

L.

#### Grossulariaceae

#### Ribes burejense

F. Schmidt

#### Ribes giraldii

Jancz.

#### Ribes komarovii

Pojark.

#### Ribes mandshuricumvar.subglabrum

Kom.

#### Ribes nigrum

L.

#### Ribes procumbens

Pall.

#### Ribes ussuriense

Jancz.

#### Haloragaceae

#### Myriophyllum spicatum

L.

#### Saxifragaceae

#### Astilboides tabularis

(Hemsl.) Engl.

#### Mukdenia acanthifolia

Nakai

#### Chrysosplenium lectus-cochleae

Kitag.

#### Chrysosplenium pilosumvar.valdepilosum

Ohwi

#### Chrysosplenium serreanum

Hand.-Mazz.

#### Saxifraga laciniata

Nakai & Takeda

#### Saxifraga manshuriensis

(Engl.) Kom.

#### Saxifraga stolonifera

Curtis

#### Solanales

#### Convolvulaceae

#### Calystegia sepium spectabilis

Brummitt

#### Cuscuta chinensis

Lam.

#### Solanaceae

#### Physalis pubescens

L.

#### Vitales

#### Vitaceae

#### Ampelopsis glandulosavar.heterophylla

(Thunb.) Momiy.

#### Ampelopsis glandulosavar.brevipedunculata

(Maxim.) Momiy.

#### Zygophyllales

#### Zygophyllaceae

#### Tribulus terrestris

L.

## Analysis

A total of 124 families, 503 species and 1640 species of tracheophytes in Xiao Hinggan Ling have been compiled. Compared with the list of “Floristics and Distribution of Plants in Xiao Hinggan Ling China”, The updated list adds 569 species, 56 genera and 6 families to the old list (Table 1, Table 2). Amongst them, there are 1 genus and 8 species of clubmosses, 6 species of gymnosperms, including 6 families, 55 genera and 555 species of angiosperm. The 569 newly added species of tracheophytes belong to 91 families and 267 genera; the results of family size ranking according to the number of species in families are shown in Table 2. The newly added families are Acoraceae, Butomaceae, Linderniaceae, Moraceae, Pedaliaceae and Zygophyllaceae and the results of ranking 56 newly added genera by genus size are shown in Table 3).

## Discussion

This study added 569 new species, 56 genera and 6 families records to the known checklist. There are 2 main reasons for the results. First, the old checklist was obtained from the IFP, which had more than 290,000 tracheophyte specimens, far less than NSII (more than 10.41 milion). Second, some new record species were found through our field survey, such as some invasive species and exotic plants (e.g.*Trifolium repens*, *Helianthus tuberosus* and *Erigeron annuus*) (Van Der Wal et al. 2008). The phenomenon may be due to the increase in the degree of human interference which provides suitable conditions for the invasion of alien plants in the Xiao Hinggan Ling area. In addition, with the warming of the climate, the plant distribution belt moves northward and some plants in the adjacent flora gradually infiltrate into the Xiao Hinggan Ling region, which is also an important reason for the emergence of some newly recorded species in this region (Parmesan and Yohe 2003).

The new list can reflect the plant varieties in the natural ecosystem of Xiao Hinggan Ling. It can provide detailed and reliable basic data for the study of flora, biodiversity and alien plant invasion in Xiao Hinggan Ling (Liu and Ji 1995).

## Supplementary Material

Supplementary material 1 New Checklist of vascular plants in Xiao Hinggan Ling-complete Data type: Checklist
File: oo_248961.xls Hongfeng Wang

## Figures and Tables

**Table 1.** Composition of the new checklist of Tracheophyte in Xiao Hinggan Ling.

Taxa	Number of families	Number of genera	Number of species	New families recorded	New genera recorded	New species recorded
Pteridophyta	16	31	67	1	2	8
Gymnosperms	3	6	18	0	0	6
Angiosperms	103	466	1555	8	65	555
Total	122	503	1640	9	67	569

**Table 2.** Tracheophyte families and their species diversity in Xiao Hinggan Ling.

Families	Number of families	Species within family
Asteraceae, Ranunculaceae, Rosaceae, Cyperaceae, Fabaceae, Poaceae, Polygonaceae, Apiaceae, Lamiaceae, Caryophyllaceae, Brassicaceae	11	≥10 species
Ericaceae, Euphorbiaceae, Plantaginaceae, Salicaceae, Saxifragaceae, Campanulaceae, Crassulaceae, Grossulariaceae, Primulaceae, Amaryllidaceae, Asparagaceae, Caprifoliaceae, Geraniaceae, Onagraceae, Papaveraceae, Pinaceae, Amaranthaceae, Betulaceae, Celastraceae, Liliaceae, Potamogetonaceae, Violaceae	22	5-10 species
Boraginaceae, Gentianaceae, Oleaceae, Orchidaceae, Orobanchaceae, Rubiaceae, Typhaceae, Adoxaceae, Araliaceae, Linaceae, Malvaceae, Rhamnaceae, Ulmaceae, Urticaceae, Alismataceae, Apocynaceae, Araceae, Asphodelaceae, Convolvulaceae, Eriocaulaceae, Fagaceae, Hydrangeaceae, Iridaceae, Juncaceae, Lythraceae, Nymphaeaceae, Ophioglossaceae, Oxalidaceae, Phyllanthaceae, Sapindaceae, Smilacaceae, Vitaceae	32	2-5 species
Acoraceae, Aristolochiaceae, Athyriaceae, Balsaminaceae, Berberidaceae, Butomaceae, Cannabaceae, Ceratophyllaceae, Cornaceae, Dennstaedtiaceae, Dioscoreaceae, Dryopteridaceae, Equisetaceae, Haloragaceae, Hydrocharitaceae, Linderniaceae, Moraceae, Onocleaceae, Phrymaceae, Plumbaginaceae, Polemoniaceae, Salviniaceae, Santalaceae, Solanaceae, Zygophyllaceae	25	1 species

**Table 3.** Tracheophyte genera and their species diversity in Xiao Hinggan Ling.

Genera	Number of genera	Size
Chrysanthemum, Ampelopsis, Arabidopsis, Arctous, Atraphaxis, Avena, Cleistogenes, Corispermum, Epipactis, Eriocaulon, Fimbristylis, Oenothera, Ophioglossum, Pennisetum, Phlomoides, Phyllanthus, Smilax, Trapa	18	≥2 species
Acorus, Ainsliaea, Alangium, Amblynotus, Anethum, Anthriscus, Arthraxon, Astilboides, Batrachium, Brachypodium, Butomus, Cannabis, Carlesia, Carpinus, Cnidium, Conyza, Desmodium, Epimedium, Eriocycla, Eritrichium, Euryale, Glycyrrhiza, Grewia, Hedysarum, Hylodesmum, Leptopyrum, Limonium, Lindernia, Lolium, Matricaria, Monotropastrum, Morus, Mukdenia, Nabalus, Osmorhiza, Ostericum, Ostryopsis, Oxytropis, Parietaria, Periploca, Physalis, Pleurospermum, Salvinia, Sibbaldia, Sibbaldianthe, Stevenia, Trapella, Tribulus, Trichophorum	49	1 species
